# Influence of Plant-Based Proteins on the Fresh and Cooked Characteristics of Ground Beef Patties

**DOI:** 10.3390/foods10091971

**Published:** 2021-08-24

**Authors:** Jase J. Ball, Ross P. Wyatt, Barry D. Lambert, Hunter R. Smith, Tristan M. Reyes, Jason T. Sawyer

**Affiliations:** 1 Department of Animal Sciences, Tarleton State University, Stephenville, TX 76402, USA; jase.ball@zoetis.com (J.J.B.); blambert@tarleton.edu (B.D.L.); 2 Archer Daniels Midland, Decatur, IL 62526, USA; ross.wyatt@adm.com; 3 Department of Animal Sciences, Auburn University, Auburn, AL 36849, USA; hzs0101@auburn.edu (H.R.S.); tzr0039@auburn.edu (T.M.R.)

**Keywords:** Allo-Kramer shear force, ground beef, oat protein, pea protein, rice protein

## Abstract

Blended meat/plant products are capturing industry market space at the retail counter for value-added beef products. Plant protein ingredients can be added to meat formulations to create appealing and functional products. Ground beef was combined with one of three plant protein inclusion treatments: control, pea, oat, or rice, along with 5% textured vegetable protein (TVP) and 1.5% soy protein concentrate then formed into 226 g patties containing up to 10% plant-based proteins. Patties were analyzed for fresh and cooked characteristics throughout a 5- or 7-day retail display. The inclusion of plant-based proteins negatively affected the instrumental tenderness values which were greater (*p* < 0.01) in plant-inclusion patties compared to the control patties. The inclusion of plant proteins increased (*p* = 0.01) the cooking yield of patties compared to the control. Cooking time was longer (*p* = 0.04) for oat patties compared to the control patties. Cooked color values for vegetable inclusion patties did not affect (*p* = 0.12) lightness (CIE L*) values; however, redness (CIE a*) was greater (*p* < 0.01) for rice than all other treatments and yellowness (CIE b*) values were greater (*p* < 0.01) for all protein treatments compared to the control. Rice improved (*p* < 0.01) fresh a* values on day 5 of display compared to the control; whereas pea decreased (*p* = 0.04) values compared to the control. There was a treatment × day interaction (*p* < 0.01) on lipid oxidation values with a reduction in values on day 3 for all vegetable proteins compared to the control and on day 7 lipid oxidation was reduced (*p* ≤ 0.03) for oat patties.

## 1. Introduction

The retail sector for plant-based foods has increased by 27% in the past year and is approaching a $7 billion market sector [1]. The landscape of the fresh meat counter is evolving as consumers modify their purchasing decisions based on improved knowledge of meat and food products. It is estimated that by 2035 the growth of alternative foods such as meat, eggs, dairy, and seafood will exceed 11% [2]. These changes in consumer awareness are providing opportunities for the meat industry to further investigate technologies for the expansion of blended meat/plant products. As the supply of beef throughout recent years has fluctuated, it has guided the meat industry to create and offer a greater percentage of inexpensive beef products and beef-based products [3]. Consumer demand for ground beef products has increased within the United States along with an increase in overall beef demand throughout developing countries [4]. Moreover, plant proteins are gaining popularity in the market segments throughout all facets of the consumer-based industries of retail and foodservice. To minimize the increase in beef pricing with varying inventories, the infusion of dietary fiber via stealth, or improve public health, it is necessary to identify technologies that could be added to the ground beef patty manufacturing process to increase formulation yields and maintain consumer acceptability [3,4,5]. Many plant-derived technologies exist that could be used as a mechanism to improve shelf-life, moisture retention, taste, texture, and tenderness, to meet consumer demand using beef trim instead of whole muscles destined for the retail counter as retail cuts [6]. The decreased beef population, in addition to reduced crop production [7], has further increased the cost of livestock production for meat animals, thereby leading to greater consumer meat prices at the retail counter or foodservice entity. Oat protein characteristics can provide greater water holding capacity which reduces the total amount of raw beef materials during ground beef patty manufacturing [8]. In addition, an increase in dietary fiber intake is considered desirable as the recommended daily intake (25 to 35 g) for the maintenance of health and prevention of diseases is rarely achieved by the population in Western countries [9]. Plant-derived proteins are considerably less expensive compared to beef trim, therefore allowing for sustained beef patty production globally [10]. One study found indicated that the inclusion of 3 to 5% of glutinous rice flour in ground beef patty formulations reduced cooking loss without negatively affecting cooked or fresh patty characteristics [11]. Furthermore, [12] demonstrated that the use of pea protein in a high-fat ground beef formulation increased fat retention and cooking yield. The inclusion of vegetable fibers in chicken burgers is an effective replacement for functional ingredients and fat for food manufacturers looking to create blended (meat/plant) products with functional ingredients that are more appealing to reducetarians or health-conscious consumers [13]. The objective of the current study was to evaluate the influence of plant-based proteins in a ground beef patty formulation and the subsequent implications on fresh and cooked beef patty surface color, yield, and textural characteristics.

## 2. Materials and Methods

Coarse ground beef (81% lean) was purchased from a commercial beef processor (National Beef, Inc., Kansas City, MO, USA), transported to the Tarleton State University Meat Laboratory, and stored at 2 °C. Coarse ground beef was allocated to one of four treatments (Control, Oat Protein, Pea Protein, and Rice Protein) in 4.91 kg batches (three batches/treatments), and plant-based proteins were manufactured by and sourced from SunOpta Ingredients (Edina, MN, USA). Each batch (4.91 kg/batch) of coarse ground beef was mixed with 3.5% of plant protein in addition to water (17%), salt (0.75%), phosphate (0.30%), textured vegetable protein (5.00%; Archer Daniels Midland, Decatur, IL, USA), soy protein concentrate (1.00%; Archer Daniels Midland, Decatur, IL, USA), and black pepper (0.25%) Coarse ground beef assigned to serve as a positive control included the same formulation with the exception of the plant protein (0%). Formulations were mixed for 5 min in a Butcher Boy ribbon mixer (Model 250M, Selmer, TN, USA). Treatment batches were then transferred to a Biro mixer/grinder (AFNG-24, Marblehead, OH, USA) and ground once through a 9.5-mm plate. Treatment batches of ground beef were then formed into 226 g patties using an automated patty former (Super 54, Model No. 65376, Hollymatic Corporation, Countryside, IL, USA). After forming, patties were randomly assigned to either Allo-Kramer shear force, cook time, instrumental cooked color, cooking yield, then labeled and individually vacuum packaged (Multivac C500, Kansas City, MO, USA) in a 20.32 cm × 38.10 cm vacuum barrier package. Packaged patties were subsequently frozen at −10 °C until analyses could be completed. Patties for instrumental fresh color, lipid oxidation, and moisture loss were placed onto a Styrofoam tray, overwrapped with a polyvinyl chloride film (PVC), and displayed for 7 days in a three-tiered (Model 60DXB-N, Turbo Air Inc., Long Beach, CA, USA) refrigerated display case operating at 4 °C with four 15 min defrost cycles every 24 h. The lighting intensity of each shelf was 2300 lux (ILT10C, International Light Technologies, Peabody, MA, USA), and storage temperatures were monitored during the display period using a recording device (TD2F, Thermoworks, American Fork, UT, USA) placed in the center of the shelf. Packages of patties were distributed evenly and rotated daily from side to side and front to back within the retail display cooler to eliminate temperature variation and simulate consumer package shifting at the retail counter.

### 2.1. Allo-Kramer Shear Force

Patties identified for instrumental tenderness (Allo-Kramer shear force) were thawed for 12 h at 4 °C and then removed from packaging prior to obtaining the Allo-Kramer shear force values [14]. Patties were cooked on an electric griddle pre-heated to 176 °C and flipped over every 2 min throughout cooking until internal temperature in the geometric center of the patty reached 71 °C [15]. Internal temperatures were monitored with a digital thermometer (Model CD28 K Type, Comark Instruments, Beaverton, OR, USA). After cooking, patties were allowed to cool to room temperature 23 °C. Allo-Kramer shear force tests were performed using an Instron Universal Testing Machine (Model 1011, Instron Corporation, Norwood, MA, USA). A standardized sample size (2 × 5 cm^2^) was cut and placed flat in a 5-blade Allo-Kramer shear cell attached to a 500 kg load cell with a standard load range setting of 100 kg and a crosshead speed of 500 mm/min. Kilograms of shear force were then converted to Newtons of force: (kg of shear force × 9.8).

### 2.2. Cooking Time and Yield

Frozen patties were thawed at 4 °C for 12 h prior to cooking. Prior to cooking, each patty was removed from their respective package, blotted dry with a paper towel, and weighed on a balance. Patties were cooked using the same procedures described above for Allo-Kramer shear force. After cooking, patties were allowed to cool to room temperature (23 °C), post-cooking weight and patty thickness were recorded. Cook yield was calculated using the following formula: [cooked weight/raw weight] × 100.

### 2.3. Instrumental Cooked Color

Prior to capturing objective surface color readings for fresh and cooked color, the colorimeter was calibrated using standard black and white tiles [16]. Frozen patties were thawed and cooked according to the cooking methods described for Allo-Kramer and Cooking Time/Yield. After cooking and cooling to room temperature (23 °C), patties were sliced through the geometric center using a Hobart slicer (Model 3813, Hobart, Troy, OH, USA). Immediately after slicing, instrumental cooked color readings were collected by scanning the internal side of each patty using a HunterLab MiniScan XE Plus (Model 45/0-L, Hunter Associates Laboratory Inc., Rustin, VA, USA). Color readings (L*, a*, b*) were recorded with an Illuminant A light source, 10° standard observer, and a 25-mm viewing aperture using the Commission Internationale de l’ Eclairage (CIE L*a*b*) color scale [17]. At random locations on the patty, three readings were measured for lightness (higher “CIE L*” value is indicative of a lighter color), redness (higher “CIE a*” value is indicative of a redder color), and yellowness (higher “CIE b*” value is indicative of a more yellow color). Instrumental cooked color values were used to calculate hue angle (representing a change from the true red axis) as: tan^−1^(CIE b*/CIE a*), chroma (representing the intensity of light) as: (CIE a*^2^ + CIE b*^2^)^1/2^; and red to brown, calculated using the reflectance ratio of 630:580 nm [16].

### 2.4. Instrumental Fresh Color

Fresh instrumental color readings were measured on each patty assigned to retail display on days 0, 3, and 5 at 1700 h. Patties were scanned three times to determine an average surface color reading (CIE L*, CIE a*, and CIE b*). Instrumental color values were used to calculate hue angle, chroma, and relative color forms of myoglobin using calculated spectral values as described above for instrumental cooked color [18]. Instrumental color readings were captured each day of the simulated display except for days 6 and 7 when instrument failure occurred.

### 2.5. Lipid Oxidation

Patties used for testing for lipid oxidation were removed from the display case on days 0, 3, and 7, vacuum packaged and frozen until further analysis. A modified version of lipid oxidation thiobarbituric acid reactive substances (TBARS) was conducted [19,20]. Prior to completing TBARS analysis, patties were thawed at 4 °C for 12 h. From each patty, 2 g of meat was homogenized (AHS 250, VMR Power Max, Radnor, PA, USA) for 60 s with 8 mL of a cold (1 °C) 50 mM phosphate buffer mix standardized to a pH of 7, containing 0.1% ethylenediaminetetra acetic acid (EDTA), 0.1% n-propyl gallate, and 2 mL of trichloroacetic acid (Sigma, St Louis, MO, USA). Samples were then filtered through filter paper (Whatman No. 4), and duplicate 2-mL aliquots of clear supernatant were then transferred into 10 mL borosilicate tubes, mixed with 2 mL of 0.02 M 2-thiobarbituric acid reagent (Sigma, St Louis, MO, USA), and boiled at 100 °C for 15 min. Immediately after boiling, tubes were placed into an ice bath for 15 min. Absorbance was measured at 533 nm with a spectrophotometer (Thermo Fisher Scientific., model Genesys 10 UV, Waltham, MA, USA) and TBARS were calculated as mg of maldonaldehyde per kg of sample [21].

### 2.6. Moisture Loss

Percent of moisture was conducted on each patty assigned to lipid oxidation analysis. On days 0, 3, or 7, patties were removed from the display case, and patties were reweighed on a balance. Moisture loss was determined using the following formula: {[Initial weight − Final weight] ÷ [Initial weight] × 100}.

### 2.7. Experimental Design and Statistical Analysis

Data were analyzed using the MIXED procedures of SAS (SAS Institute, Inc., Cary, NC, USA). All data were analyzed as a randomized complete block design with a beef patty serving as the experimental unit and blocked by batches within the treatment. Analysis of variance was generated with treatment as the lone fixed effect, and a block as the lone random effect, while patty replication was used as a repeated measure for moisture retention, instrumental color, cook yield, moisture retention, and instrumental tenderness. Least squares means were generated, and, when significant (*p* ≤ 0.05) F-values were observed, least squares means were separated using a pair-wise *t*-test (PDIFF option).

## 3. Results and Discussion

### 3.1. Allo-Kramer Shear Force

Allo-Kramer shear force values were greater (*p* < 0.01) for patties formulated with the plant-based proteins compared to the control patties (Table 1) suggesting that plant-based protein inclusion negatively affects the instrumental tenderness. Results from this study differ from other studies that suggested that oat protein had no effect on the instrumental tenderness values [22]. It is plausible that the combination of added protein, salt, and water caused an increase in moisture retention resulting in a rigid patty. Unfortunately, neither compression analysis nor sensory evaluation were performed which could have provided additional information on the changes in patty texture. It is important to note that previous studies on ground beef patty shear force were lower as the percentage of fat content in the patty increased [22]. The addition of fat likely caused a lubrication effect on the mechanical shear force. It is interesting to note that globally, many consumers are searching for leaner and healthier food options when completing retail purchases, so consumers may overlook a decrease in tenderness for enhanced beef patties with the inclusion of plant-based proteins [23]. The addition of plant-based proteins within ground beef patty formulations could be perceived by consumers as a solution for blended proteins for consumers who are more health-conscious, which agrees with previous studies where the addition of oat protein and other fat replacers can decrease the caloric content of ground beef patties from 790 to 585 kilojoules [22]. Nonetheless, patty texture could be an influencer of consumer appeal during the eating experience and should be considered when considering the inclusion of a plant-based protein.

### 3.2. Cooking Time

Cooking time increased (*p* = 0.04) with the use of the oat protein when compared to the control; however, no other proteins affected cooking time (Table 1). The oat protein required a > 22 s time to reach an internal core temperature of 71 °C compared to all other treatments. The current results for cooking to an endpoint temperature agree with previous findings, which suggest that the addition of the pea protein within a ground beef application appeared not to alter the endpoint temperature of ground beef patties [24]. The formulation of ground beef patties with plant-based proteins has inconsistently altered the cooking time of ground beef patties. This variance in cooking time differs from the findings in our previous study using different inclusion rates of the oat protein [25]. Adjustments in cooking time should be evaluated by restaurants and manufacturers of ground beef patties when using meat/plant blended patties. However, variations in cooking time may be attributed to differences in patty weight, fat content, moisture retention, and the cooking state (fresh vs. frozen).

### 3.3. Cooking Yield

For the meat industry producing value-added products, the emphasis tends to be placed on cooking yield to increase profit margins along with gaining customers who appreciate a juicier and more flavorful product. Unfortunately, sensory evaluation was conducted during the current study in conjunction with the analysis for cooked and fresh characteristics. For this study, the cooking yield was greater (*p* = 0.01) for patties containing plant-based proteins than for the control patties (Table 1). Results suggest that the inclusion of plant-based proteins in ground beef patty formulations will improve cooking yield by retaining more moisture during the cooking process. However, there were no differences in cooking yield between the different plant proteins indicating that the source of the protein inclusion may not affect the cooking yield. The results from cooking yield percentages coincide with previous research which suggested that an increase in protein within the meat block formulation increased the cooking yield percentages for ground beef patties [24]. Increases in the percent of cooking yield are derived from a greater percentage of moisture retained during cooking, therefore, potentially resulting in a reduction of costs associated with beef trim used during product formulations [24]. A decrease in cooked weight in relation to the raw fresh weight can also be associated with moisture loss through a loss of fat during the cooking process, which can vary with regards to the meat block formulation [26]. With this increase in the percent of cooking yield, the presence of a plant-based protein within a ground beef formulation could further extend the limited supply of beef, thus, reducing manufacturing costs. Ground beef studies conducted suggest that, as the fat content is reduced from 25–30% to 5–10%, cooking loss, drip loss, juiciness, beef flavor, tenderness, oily mouth-coating, and consumer demand often decreases [27,28,29,30,31]. These results indicate that the utilization of plant-based proteins will impart deleterious impacts on some attributes but does improve cooking yields. A study hypothesized that the mechanism allowing the increase in cook yield and increased moisture retention may be physical in nature [13]. The swelling of the plant-based proteins within ground beef patties may interact with the protein from the beef patty to form a matrix, which acts to prevent the coalescence and migration of fat and water out of the product.

### 3.4. Instrumental Cooked Color

Vegetable-based proteins influenced the instrumental cooked color of ground beef patties (Table 2). This can be important as many retailers and foodservice operators aim to market fully cooked patties and changes in either cooked or fresh surface color could potentially have a negative impact on consumer preference at the time of purchase or consumption. The inclusion of a vegetable protein did not affect (*p* = 0.12) lightness (CIE L*) values. Rice increased (*p* < 0.01) redness (CIE a*) values compared to the control, oat, and pea. This suggests that rice may have a greater effect on cooked redness in ground beef patties compared to other plant-derived proteins. Yellowness (CIE b*) values increased (*p* < 0.01) with the inclusion of plant-based proteins compared to the control. Furthermore, rice had the greatest (*p* < 0.01) yellowness value followed by pea and then oat of the ground beef patty formulations with plant-derived proteins. Oat and pea improved (*p* < 0.01) hue angle values compared to the control. The inclusion of rice and pea increased (*p* < 0.01) chroma values compared to oat and the control indicating greater total color intensity and suggesting that rice and pea proteins impeded the denaturation of myoglobin during the cooking process of the patties. The instrumental cooked color evaluation of red to brown (630:580 nm) in ground beef patty formulations with the inclusion of plant-derived fibers was greater (*p* < 0.01) for rice compared to the control suggesting that patties with the inclusion of the rice protein were redder after cooking. Previous research reported that there was no difference in internal cooked color for ground beef patties with varying lean-to-fat ratios [32]. These previous findings, along with the results from our laboratory oat protein study, suggest different lean-to-fat ratios along with varying percentages of vegetable-based proteins in the meat block could alter the internal cooked color of ground beef patties. Other factors that could lead to the influence of the internal cooked color could be associated with variations in the total myoglobin of patties containing a greater percentage of non-meat ingredients. This tends to agree with a study that suggests other factors such as processing parameters and non-meat ingredients can influence the internal color of meat items following a cooking process [33].

### 3.5. Instrumental Fresh Color

**Figure 1:**
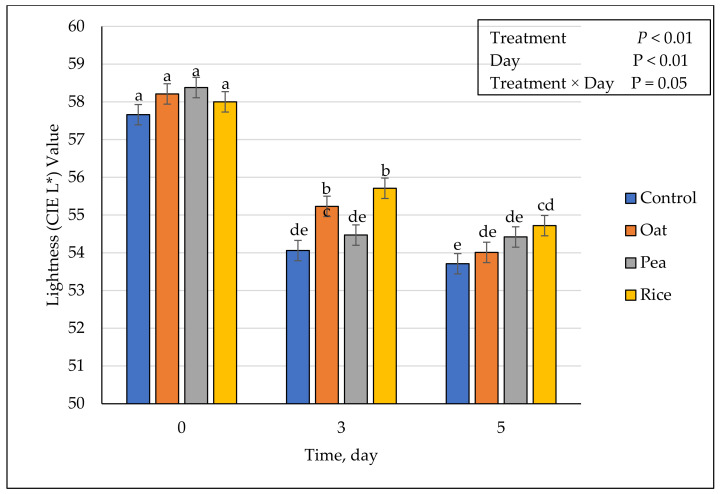
Effect of vegetable-based protein inclusion in ground beef patty formulation on lightness (CIE L*) values during simulated retail display. Bars lacking common superscripts differ (*p* ≤ 0.05).

**Figure 2:**
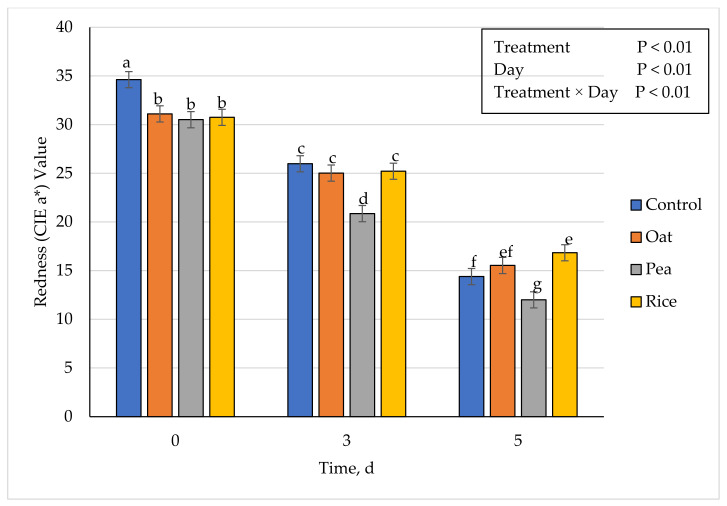
Effect of vegetable-based protein inclusion in ground beef patty formulation on redness (CIE a*) values during simulated retail display. Bars lacking common superscripts differ (*p* ≤ 0.05).

**Figure 3:**
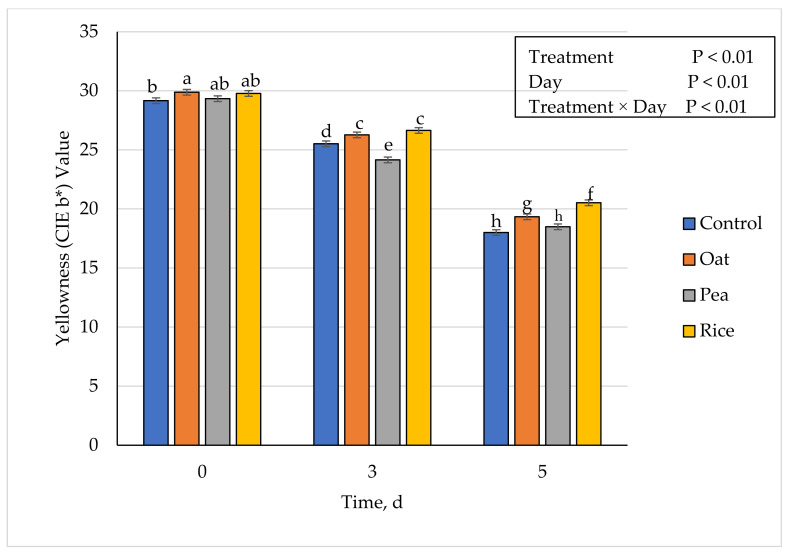
Effect of vegetable-based protein inclusion in ground beef patty formulation on yellowness (CIE b*) values during simulated retail display. Bars lacking common superscripts differ (*p* ≤ 0.05).

**Figure 4:**
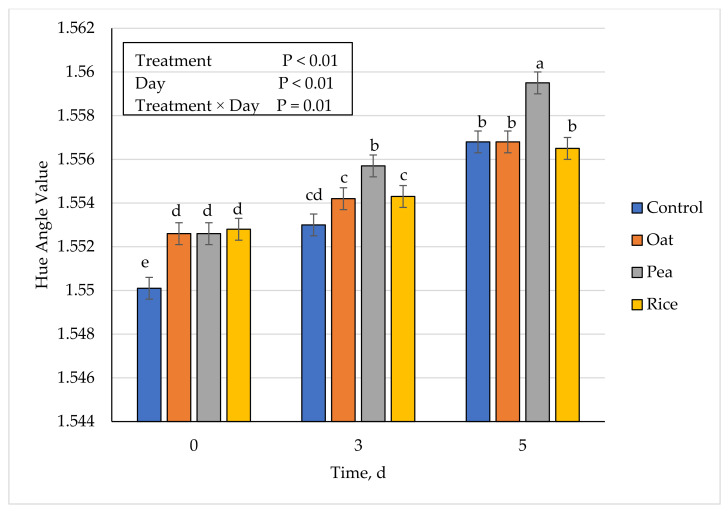
Effect of vegetable-based protein inclusion in ground beef patty formulation on hue angle values during simulated retail display. Bars lacking common superscripts differ (*p* ≤ 0.05).

**Figure 5:**
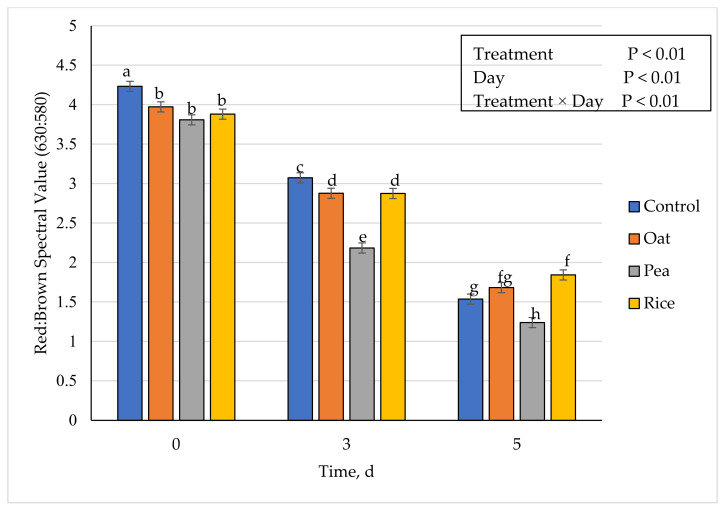
Effect of vegetable-based protein inclusion in ground beef patty formulation on oxymyoglobin values during simulated retail display. Bars lacking common superscripts differ (*p* ≤ 0.05).

**Figure 6:**
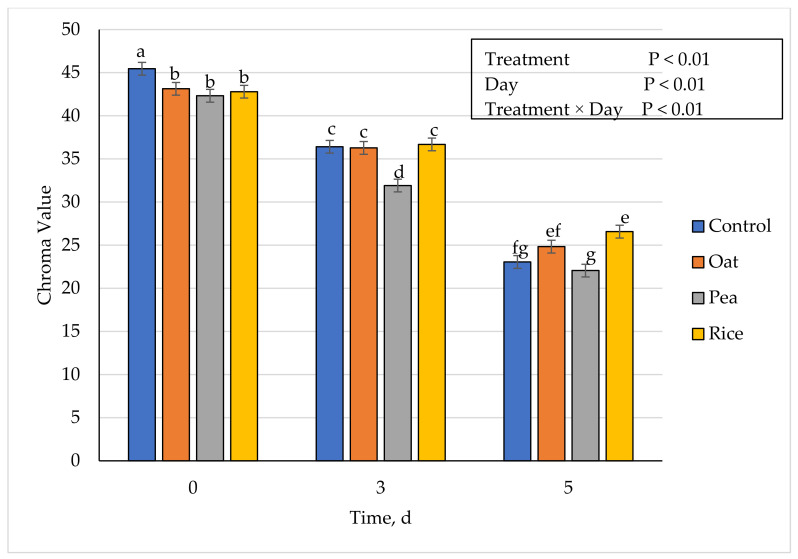
Effect of vegetable-based protein inclusion in ground beef patty formulation on chroma values during simulated retail display. Bars lacking common letters differ (*p* ≤ 0.05).

There was a treatment × day interaction (*p* = 0.05) for lightness (CIE L*) values (Figure 1), in which values (CIE L*) for fresh ground beef patties, regardless of treatment, decreased during simulated retail display. On days 3 and 5, lightness values were greater (*p* < 0.01) for rice compared to the control. The increase of fresh color lightness with the inclusion of rice coincides with cooked color lightness results, suggesting rice is the protein source of the greatest effect on lightness in patty formulations of all protein sources evaluated. A treatment × day interaction was detected (*p* < 0.01) for fresh redness values (CIE a*; Figure 2). On day 5 of the simulated retail display, rice increased (*p* = 0.04) redness values compared to the control. The improvement of redness at the end of the simulated retail display with the inclusion of rice in the ground beef patty formulation is similar to the results seen in the cooked color evaluation. The decrease in redness (CIE a*) values for all treatments during the simulated retail display resulted in a ground beef patty with a darker surface color, similar to results in beef steaks and ground beef [34,35,36,37]. There was a treatment × day interaction (*p* < 0.01) for fresh yellowness values (CIE b*; Figure 3). On days 3 and 5, rice and oat improved (*p* ≤ 0.03) fresh yellowness values compared to the control. A treatment × day interaction for fresh hue angle values was detected (*p* = 0.01) during the simulated retail display (Figure 4). Fresh surface color hue angle values were greater (*p* < 0.01) for pea compared to the control on days 0, 3, and 5 of retail display. Furthermore, pea values for hue angle were greater (*p* ≤ 0.04) than all other fibers on days 3 and 5. Hue angles with a larger value are indicative of a less red (cooked or fresh) color. A treatment × day interaction occurred (*p* < 0.01) for red to brown (630:580) content within the fresh ground beef patties (Figure 5). On day 5 of the retail display, rice increased (*p* < 0.01) red to brown values compared to the control; whereas pea decreased (*p* < 0.01) red to brown values compared to the control. It appears that by day 5, the rice protein imparted an influence on the instrumental surface color red to brown content of patties which coincides with fresh redness value deterioration during the simulated display settings. Spectral values have been used during the simulated display periods to capture changes in the surface color from red to brown (630:580). However, this value is less specific for capturing oxymyoglobin of a fresh meat surface [16] due to the redness associated with deoxymyoglobin. A treatment × day interaction was detected (*p* < 0.01) for chroma values during simulated retail display (Figure 6). Fresh chroma values were increased (*p* < 0.01) on day 5 of the simulated retail display with the inclusion of rice compared to the control. Research showed that when myoglobin content is constant, the color of comminuted products is mostly influenced by processing parameters, fat content, non-meat ingredients, and added or lost water [33]. Instrumental fresh surface color values suggest that the inclusion of plant-based proteins alters the total myoglobin quantities as non-meat ingredient inclusion increases within a meat formulation. The interactions for many instrumental fresh color values in this study are plausibly due to changes in myoglobin content that occurred because of the manufacturing of the blended patties and, subsequently, influenced the surface color of the patties throughout the display period. Of the proteins evaluated, rice had the least detrimental impact on fresh color characteristics compared to the control whereas pea resulted in only slight changes to fresh color characteristics and there were minimal differences with the inclusion of oat.

### 3.6. Lipid Oxidation (TBARS)

**Figure 7:**
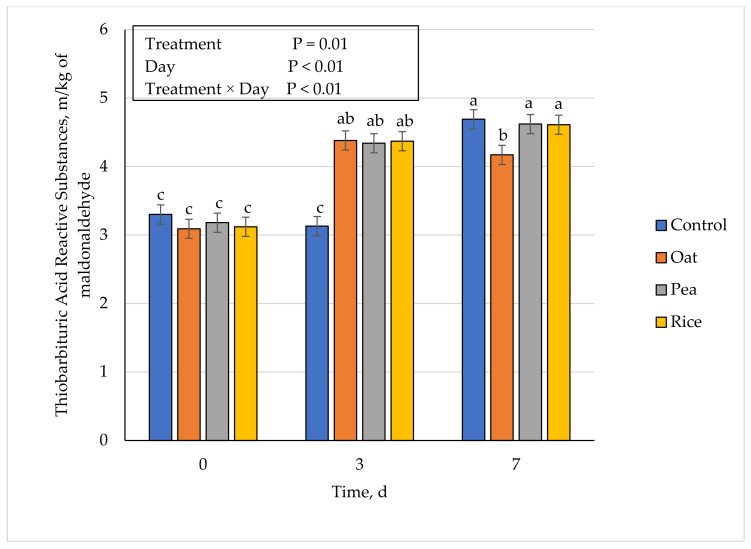
Effect of vegetable-based protein inclusion in ground beef patty formulation on lipid oxidation values during simulated retail display. Bars lacking common letters differ (*p* ≤ 0.05).

A treatment × day interaction occurred (*p* < 0.01) for lipid oxidation values in ground beef patties containing a plant-based protein (Figure 7). On day 0, there were no differences (*p* ≥ 0.35) across patty formulation treatment, suggesting that plant-based proteins do not have a major effect on lipid oxidation immediately after manufacturing. However, by day 3, there was a significant increase (*p* < 0.01) in lipid oxidation values for patties containing plant-based proteins when compared to the control patties. Finally, on day 7 of the simulated retail display, lipid oxidation was less (*p* ≤ 0.03) for oat compared to all other treatments including the control, suggesting that oat protein might have the potential to impact lipid oxidation values. There were no differences (*p* ≥ 0.68) between pea, rice, and the control on day 7 of the simulated retail display for lipid oxidation values. However, additional studies should be conducted further evaluating the impact of plant-based protein on lipid oxidation in a ground beef formulation. The results from this study differ from the one which concluded that adding legume flours to a meat formulation did not have an effect on lipid oxidation during retail display [38]. Lipid oxidation is one of the important limiting factors for the quality and acceptability of meat and meat products [39]. Furthermore, lipid oxidation has been reported to increase with increasing storage periods during refrigerated display [40]. Moreover, studies have reported TBARS values ranging from 0.6 to 2.0 mg maldoaldehyde/kg [41,42]. The degradation of lipids throughout the display period was altered by the presence of a vegetable-based protein, more specifically, the use of an oat protein within an industry setting for ground beef patties could be beneficial to enhancing shelf-life. These lipid oxidation results could further enhance the marketability of a plant-based protein application in ground beef patties, which could potentially minimize the number of markdowns and throwaways that retail providers encounter.

### 3.7. Moisture Loss during Simulated Retail Display

A treatment × day interaction was not detected (*p* = 0.10) for moisture loss evaluated during the duration of a 7-day simulated retail display (Table 3). There was a main effect of treatment where moisture loss was greater (*p* ≤ 0.02) for rice compared to oat and pea; however, no differences (*p* ≥ 0.16) were noted between the control and any of the plant protein treatments. As expected, moisture loss increased with the greatest (*p* ≤ 0.01) loss being recorded at the end of the simulated display period. These results suggest that plant-based proteins impart a minimal impact on the amount of moisture lost during a retail display period as expected. However, previous research conducted reported that oat protein and tapioca starch both increase moisture retention in ground beef patties which agrees with this study’s findings [43]. In addition, the current results differ from previous results which reported that adding oat protein as a source of beta-glucan (13.45%) to low-fat (<10%) beef patties improved moisture retention compared to 20% fat control patties [44]. Research suggests that the addition of rice protein increases moisture retention in ground beef patties [12]. The variations in moisture loss for fresh patties from previous results could be attributed to storage temperature, the packaging method, duration of display, and patty formulation. Although differences reported for moisture loss during the simulated retail display were minimal, all patty formulations including the control resulted in a small percentage of moisture reduction.

## 4. Conclusions

The results from the current study suggest that plant-based proteins could be utilized in ground beef patty formulations without causing extensive negative effects to textural characteristics, and plausibly reduce the amount of beef trim utilized within ground beef patty manufacturing. Although texture values such as instrumental tenderness increased with the use of the plant proteins, it appears that the improvements in cooking yield and moisture retention could enhance the marketability of using plant-based proteins in a ground beef patty formulation. Of the protein evaluated, rice protein had the greatest effect on fresh and cooked color characteristics of the ground beef patty formulations. The addition of a plant-based protein within ground beef patties may extend the supply of available beef trim and allow ground beef manufacturers an alternative ground beef product combining beef- and plant-derived products to create a final product that is potentially more appealing to consumers. However, additional information is necessary for identifying the sensory taste issues that could arise from the inclusion of plant-based proteins in a ground beef application. Focused research on trained and consumer sensory evaluation could provide the additional information necessary for the consumer adoption of plant-based proteins within a ground beef formulation.

## Figures and Tables

**Table 1:** Effects of vegetable-based protein inclusion in ground beef patty formulation on Allo-Kramer shear force, cook yield, and cook time.

	Protein Treatment					
Item	Control	Oat	Pea	Rice	SEM *	*p* Value
Shear force, N	356.91 ^c^	490.96 ^b^	466.58 ^b^	538.94 ^a^	10.4	<0.01
Cook time, s	589.71 ^b^	632.74 ^a^	605.44 ^a,b^	610.01 ^a,b^	10.4	0.04
Cook yield, %	85.23 ^b^	87.87 ^a^	87.34 ^a^	88.55 ^a^	0.7	0.01

^a–c^ means within a row lacking a common superscript letter differ (*p* ≤ 0.05). * SEM, Standard Error of the Mean.

**Table 2:** Effects of vegetable-based protein inclusion in ground beef patty formulation on instrumental cooked color.

	Protein Treatment					
Item	Control	Oat	Pea	Rice	SEM	*p* Value
Lightness (CIE L*) ^1^	57.54	57.14	57.13	57.41	0.10	0.12
Redness (CIE a*) ^2^	14.74 ^b^	14.20 ^b^	15.00 ^b^	16.03 ^a^	0.30	<0.01
Yellowness (CIE b*) ^3^	15.03 ^d^	15.86 ^c^	16.38 ^b^	17.13 ^a^	0.10	<0.01
Hue Angle ^4^	1.5537 ^b^	1.5552 ^a^	1.5548 ^a^	1.5545 ^a,b^	0.01	<0.01
Chroma ^5^	21.06 ^c^	21.30 ^c^	22.22 ^b^	23.48 ^a^	0.30	<0.01
Red to Brown ^6^	1.74 ^b,c^	1.66 ^c^	1.80 ^b^	1.94 ^a^	0.05	<0.01

^1^ Lightness (CIE L*) values are a measure of darkness to lightness, a larger value indicates a lighter color (0 = black, 100 = white). ^2^ Redness (CIE a*) values are a measure of redness, larger values indicate a redder color (−60 = green, +60 = red). ^3^ Yellowness (CIE b*) values are a measure of yellowness, a larger value indicates a more yellow color (−60 = blue, +60 = yellow). ^4^ Hue angle represents the change from the true red axis with a greater value indicating a greater shift from red to yellow (Hue angle = tan^−1^ CIE b*/CIE a*). ^5^ Chroma is a measure of the total color (larger value indicates more total color). ^6^ Red to Brown is a ratio of 630:580 nm which represents a change in the color of red to brown (larger value indicates a redder color). ^a–d^ means within a row lacking a common superscript letter differ (*p* ≤ 0.05).

**Table 3:** Effects of plant-based protein inclusion in ground beef patty formulation on moisture loss ^1^ under simulated retail display.

	Treatment					*p*-Values		
	Control ^a,b^	Oat ^b^	Pea ^b^	Rice ^a^	SEM *	Treatment	Day	Treatment × Day
Day 0 ^c^	0.43	0.43	0.44	0.43				
Day 3 ^b^	1.13	0.94	0.95	1.06				
Day 7 ^a^	1.65	1.63	1.60	1.99	0.08	0.05	<0.01	0.10

^1^ Moisture Loss = (100 − percent moisture retention). ^a–c^ Means lacking a common superscript letter within row (Main effect of Treatment) or column (Main effect of Day) differ (*p* ≤ 0.05). * SEM, Standard Error of the Mean.
